# A Bibliometric and Comprehensive Review on Condition Monitoring of Metal Oxide Surge Arresters

**DOI:** 10.3390/s24010235

**Published:** 2023-12-31

**Authors:** Tiago Goncalves Zacarias, Wilson Cesar Sant’Ana

**Affiliations:** 1 Office of Research and Graduate Studies (PRPPG), Federal University of Itajuba, Itajuba 37500-903, MG, Brazil; tiagogz@live.com; 2 R&D Department, Gnarus Institute, Itajuba 37500-052, MG, Brazil

**Keywords:** condition monitoring, literature review, metal oxide surge arrester

## Abstract

This paper presents a literature review on the subject of Condition-Based Maintenance of surge arresters. Both a bibliometric analysis and traditional comprehensive research are presented. The bibliometric analysis is useful for obtaining insights about the literature. It quantitatively highlights relationships between journals, authors and keywords (related to the monitoring methods) and reveals future trends for research based on the timeline of the keywords. The traditional comprehensive literature review is also presented. It summarizes the methods, their advantages and disadvantages and also points to some known measurement issues of the methods. Both online (leakage current, harmonic components, temperature, partial discharges, power loss and the counting of discharges) and offline (reference voltage, residual voltage, insulation resistance, polarization/depolarization, return voltage, microscopy, spectrometry, X-ray, RUS and the recent application of FRA) methods have been qualitatively analyzed.

## 1. Introduction

The proper functioning of an electrical power system depends on the correct operation of all its components. A substantial cause for failures on the power grid is lightning [1,2]. It is reported that lightning strikes have caused some of the largest blackouts in history [3]. Devices have been developed over the years to overcome the damage caused by lightning, providing an alternative pathway for the discharge. In the early years of transmission systems (until the 1900s), gaps were the only known surge protection device. A series of developments in non-linear resistors (still combined with gaps) used until the end of the 1960s have been reported by [4], including the highlighted Silicon Carbide (SiC) varistor. The next evolutionary development was the gapless Zinc Oxide (ZnO) varistor. In fact, ZnO is a major component in polycrystalline semiconductors (other metal oxides, such as $Bi_{2}O_{3}$, $MnO_{2}$ and $Sb_{2}O_{3}$ are used as minor additives in intergranular spaces), and [5]—this is the origin of the term Metal Oxide Surge Arrester (MOSA).

Like each and every protective equipment, the lightning arrester is susceptible to failures and degradation, given its actions to protect the system. Hence, it becomes important to monitor its operational status to guarantee the reliability of the system [6]. The literature presents several methods and devices aiming to detect failure and degradation in arresters. Briefly, the most traditional offline techniques (where the arrester must be removed from operation, or sometimes even destroyed) are the reference voltage, residual voltage, insulation resistance, polarization/depolarization, return voltage, microscopy, spectrometry and X-ray, whereas the most traditional online techniques (where the arrester is tested without being removed from operation) are based on the monitoring of the leakage current, harmonic components, temperature, partial discharges, power loss and the counting of discharges.

Some good literature reviews/surveys have been published throughout the years. Mardira et al. in 2005, [7] presented an investigation of several offline techniques. The state-of-the-art review written by Woodworth in 2012 [8] was more focused on commercial equipment for both online and offline methods. Abdul-Malek et al. in 2015 [9] presented a review with emphasis on the aging behavior of the arresters. Nithin Reddy and Subba Reddy in 2021 [5] focused on degradation methods (in order to accelerate aging for laboratory tests). In 2022, Ranjbar et al. [10] presented a survey on both online and offline methods. However, recent [10] research did not cover methods such as Resonant Ultrasound Spectroscopy (RUS), nor a newer development that was proposed after it, which is the use of Frequency Response Analysis (FRA, a method used mainly for detection of deformation of windings in power transformers) on arresters.

This paper presents an up-to-date literature review on the methods of condition monitoring of lightning arresters, both offline and online. Apart from the monitoring methods, the underlying parameters that are going to be monitored are also discussed. The paper also innovates, presenting, for the first time in the field of condition monitoring of arresters, a bibliometric analysis.

According to Elomari et al. [11], a traditional literature review focuses on summarizing the key results based on extensive research in the literature, whereas bibliometric analysis is based on extracting the most noteworthy data from databases. This paper presents both the traditional extensive research on the literature of arresters and, also, the bibliometric analysis performed in the Web of Science (WoS) database. WoS has been prioritized over Scopus, since it returns data from all major publishers in Electrical Engineering (such as IEEE, MDPI, Elsevier, etc.). The outcome of this paper is to provide both the technical aspects of the methods (benefiting both academic researchers and industry personnel) and insights about the research field itself (benefiting policymakers, funding agencies and researchers interested in collaborations).

The paper is structured as follows: Section 2 presents an overview of the parameters related to degradation in arresters. Section 3 presents the literature review, divided in two parts. The first part (Section 3.1) presents the bibliometric analysis, with the objective of obtaining quantitative insights from the published literature on condition monitoring of arresters. The second part (Section 3.2) presents the traditional comprehensive literature review, with the objective of obtaining the qualitative aspects of the methods available.

## 2. Materials and Methods

According to Keith Mobley, in the introductory chapters of the book [12] [p. 36], condition monitoring is a management technique based on the regular evaluation of the actual operating condition of an equipment/system in order to optimize its operation. Its application in surge arresters addresses the monitoring of electrical and physical parameters. Monitoring these parameters during the surge arrester’s operating cycle can provide a lot of information about its protective capacity and reliability. The most important parameters that are related to degradation and failure of surge arresters are the leakage current (Section 2.1), insulation resistance (Section 2.2), reference voltage (Section 2.3), residual voltage (Section 2.4), temperature (Section 2.5), partial discharges (Section 2.6), power losses (Section 2.7), superficial pollution (Section 2.8), dielectric polarization (Section 2.9), number of discharges (Section 2.10), and microstructure (Section 2.11). This section provides an overview of these parameters, whereas in Section 3.2, the actual monitoring methods for these parameters are reviewed.

### 2.1. Leakage Current

**Figure 1:**
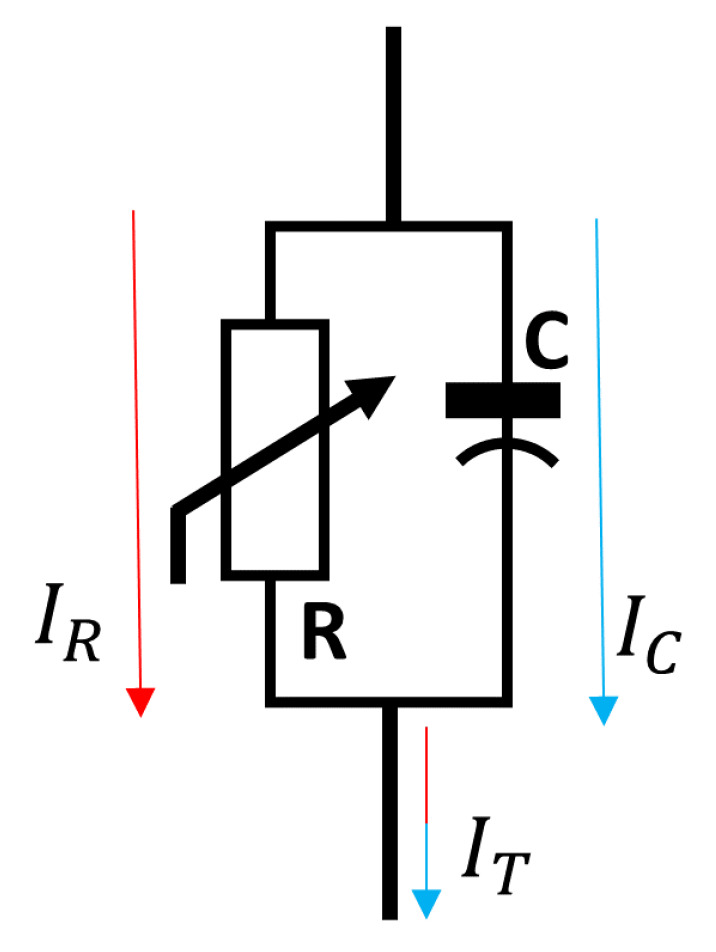
Basic equivalent circuit of a MOSA—based on [13,15].

Figure 1 presents the basic equivalent circuit of a metal oxide arrester, which is composed of a parallel resistive–capacitive association. The resistive part presents a non-linear behavior dependent on the voltage level and frequency. Under regular working conditions, the arrester behaves similarly to an insulator (presenting a small capacitive leakage current in the order of few mA [4]) and, during a surge, it conducts the surge current. A good arrester should return to its original condition after the surge; however, the resistive component is known to be directly related to the degree of degradation [13]. The total leakage current $I_{T}$ is obtained from Equation (1). Usually, the contribution of the resistive leakage current $I_{R}$ is equivalent to 5–20% of the total current [13], under normal operation, whereas typical values of the capacitive leakage current range from 0.5 to 3 mA${}_{peak}$ [14]. As an intrinsic behavior, the arrester, due to its non-linearity, produces current harmonics (in particular, the third-order harmonic component). This third harmonics component, in particular, increases in the face of degradation.

(1) $$I_{T}=I_{C}+I_{R}\;.$$

### 2.2. Insulation Resistance

The insulation resistance is a characteristic measured for elements with dielectric behavior. It is defined by the IEEE Std 43-2013 [16] as “the capability of the electrical insulation of a winding to resist direct current” (referring to the measurement of the insulation resistance of electric machinery). Extending this definition to a more general concept, it represents the ohmic impedance seen by a given electrical reference signal (either DC or AC).

### 2.3. Reference Voltage

The reference voltage is a parameter strongly related to the state of degradation of MOSAs. It relates to the application of a known and controlled voltage to the arrester terminals (with the lowest possible harmonic content) and identification of the potential, which was necessary to generate a leakage current in the order of 1 mA [7]. The variation in the reference voltage value indicates the degradation of the arrester’s internal varistor blocks (a variation of ±5% is tolerated according to [7] and ±5% to 10% according to [17]).

### 2.4. Residual Voltage

The residual voltage (also known as the discharge voltage in the USA) is the peak voltage that appears at the arrester during the passage of the surge current [18]. The measurement of this parameter is performed offline, due to the need to remove the power supply and to use impulse generators. The impulse waveform is guided by the IEEE Std C62.11-2020 [19] and the 8/20 μs current waveform is adopted. The method uses a pass/fail condition [7].

### 2.5. Temperature

**Figure 2:**
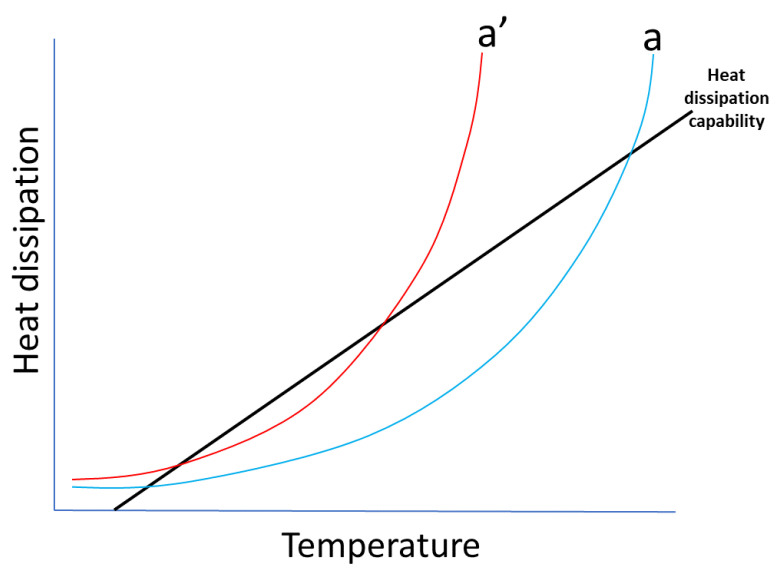
MOSA thermal characteristics diagram—based on [21].

The operating temperature of the MOSA is an indicator of its operating state. The elevation of its temperature is one of the main effects of some types of degradation [20]. The loss of the arrester’s heat exchange capacity is called a loss of thermal stability. According to [21], as illustrated in Figure 2, the increase in voltage applied to the arrester terminals or the degradation of its elements causes a reduction in its heat dissipation capability. In the figure, the blue curve (a) refers to an arrester in good condition, while curve (a’) represents this arrester in a degradation condition. The straight line represents the heat dissipation capability of the arrester, at the points where it crosses the curves. It can be noticed that the heat dissipation capability at condition (a’—degradation) is displaced to a lower value than at condition (a—good).

Due to the circulation of the leakage current and operational or environmental factors, the arrester begins to dissipate power and its electrical properties are degraded, increasing its working temperature. The resistive component ($I_{R}$) is related to power loss and temperature rise of the arrester [22,23,24]. This results in a thermal avalanche, a condition in which the arrester fails or even explodes.

### 2.6. Partial Discharges

**Figure 3:**
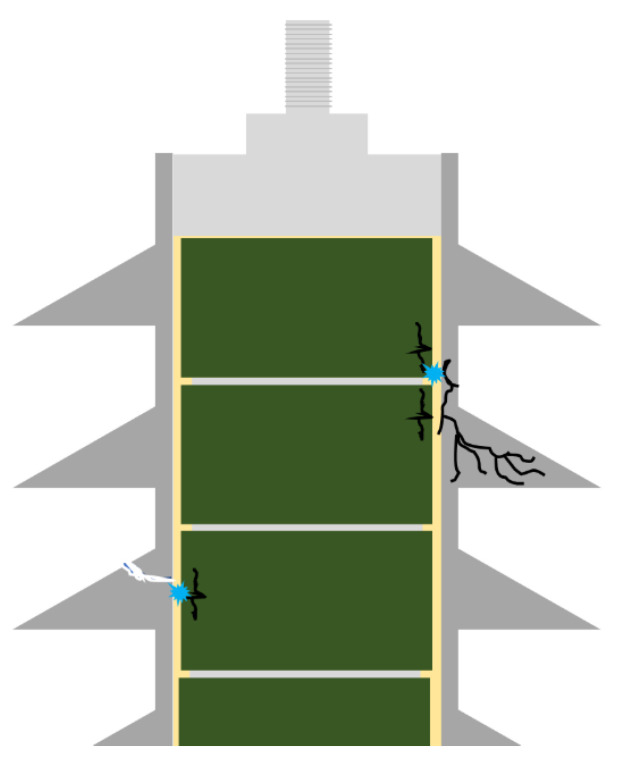
Partial discharge between ZnO blocks and housing surface.

Partial discharge is a type of failure that can compromise the condition of insulators [25]. It is defined by the IEC 60270 standard [26] as a “localized electrical discharge that only partially bridges the insulation between conductors”. In arresters, due to their behavior as a dielectric under normal conditions and when subjected to electric fields applied between their terminals, partial discharge becomes a problem. Conditions imposed on arresters in the field allow partial discharges to occur, compromising the impermeability of the housing [27]. The discharges that occur between the varistor column and the housing surface (as presented in Figure 3) are the most dangerous, as they create openings in the housing and allow moisture to enter [27].

### 2.7. Power Losses

Power loss is an indication of excessive circulation of resistive leakage current and results in an increase in the temperature of the object. Arresters are susceptible to power loss, caused inside or through the surface of the arrester. The power loss is directly proportional to the increase in the resistive component of the leakage current [28]. The power loss in polymeric-type arresters depends on internal losses (from the varistor arrangement itself) and on the loss due to pollution of the polymeric casing [29].

### 2.8. Superficial Pollution

The exposure of the arrester to the deposition of environmental pollutants can induce its premature failure [30]. The superficial pollution reduces the operational life of the arrester and intensifies failures such as partial discharges and loss of power.

### 2.9. Dielectric Polarization

When a dielectric material is subjected to an external electric field, its charges tend to line up with the field [31] [p. 734]. This process of alignment is called polarization [32] [p. 501]. The degree of polarization of a material (i.e., the more easily the material can be polarized) is related to its relative permittivity and dielectric constant [33]. Also, as the frequency of the field increases, the polarization mechanisms become less effective (resulting in dielectric losses) [33].

### 2.10. Surge Counting

With each discharge and overvoltage event, the arrester undergoes a brief period of stress, where high values of discharge current circulate. The changes caused are the sudden increase in temperature [34] and degradation of the varistor blocks internal to the arrester. Monitoring the number of discharges can be carried out by counting these sudden temperature variations [34] or by analyzing the discharge current [35].

### 2.11. Microstructure

When degradation occurs, the ZnO material undergoes changes in its crystalline structure, which directly affect its electrical properties. The behavior and reliability of the varistors are dependent on their microstructure. Differences in grain size, spinel precipitations and pores result in weaknesses in the microstructure [36]. According to [37], the conduction of elevated surge currents causes shrinkage of ZnO grains and modification of grain boundaries, affecting the electric parameters. Usually, the methods used to analyze the microstructure are destructive methods, requiring the sample to be disassembled.

## 3. Results and Discussion

The results from this literature review have been divided into two parts. The first part (Section 3.1) presents a Bibliometric analysis on the subject of Condition Monitoring of arresters. This a quantitative analysis which uses software tools on bibliographic databases in order to obtain insights from the studied subject prior to reading any of the articles. This type of analysis is usually used to uncover emerging trends in the subject, to detect what journals would be more appropriate for publishing in that subject and to highlight collaboration patterns [38]. However, it is important to note that this type of analysis is a result produced by computer software. It serves to provide an overview of a study field, although a more in-depth and traditional literature review is still required.

The second part of the results (Section 3.2) presents the in-depth and traditional literature review.

### 3.1. Bibliometric Analysis

According to [11], the search query is one of the most important stages for a bibliometric study. There are particularities that depend on the chosen database, although boolean operators (AND, OR and NOT) are usually used in most databases. Below, we present some search queries to be used with the WoS database. Terms within quotes (“”) are considered an exact phrase. Also, the wildcard * can be used to obtain different combinations of letters that could appear after the * mark (for example, diagnos* would return diagnostic, diagnostics, diagnose, diagnosis and so forth).

(2) $$\begin{array}{cc} & TS=((“condition”\;OR\;“CBM”\;OR\;“predicti*”\;OR\;“detection”\;OR\;“diagnos*”\;OR\;“assessment”)\\ & AND(“MOSA”\;OR\;“arrester*”))\;. \end{array}$$

The query of Equation (2) (performed in WoS in 19/July/2023) returned 524 papers. However, some of them were not at all related to surge arresters. The solution was to refine the query, excluding some terms using the boolean operator NOT.

(3) $$\begin{array}{cc} & TS=((“condition”\;OR\;“CBM”\;OR\;“predicti*”\;OR\;“detection”\;OR\;“diagnos*”\;OR\;“assessment”)\;AND(arrester*)\\ & NOT(“crackarrester*”\;OR\;“mitot*arrester*”\;OR\;“detonationarrester”\;OR\;“flamearrester*”\\ & \;\;\;\;\;\;\;\;\;\;\;\;OR\;“histologicalarrester*”\;OR\;“deflagrationarrester*”\;OR\;bedding))\;. \end{array}$$

**Figure 4:**
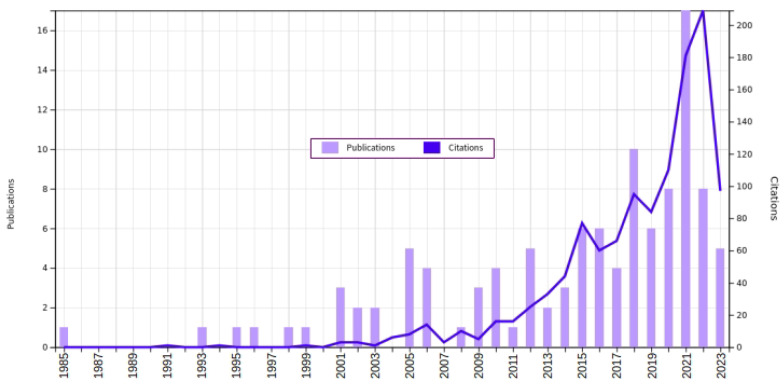
Evolution per year in the number of publications and citations related to CBM of surge arresters—Figure generated in the Web of Science website.

The query of Equation (3) (performed in WoS in 19 July 2023) returned 430 papers (divided as 231 proceedings papers, 219 articles, 2 reviews and 1 early access paper). In order to reduce the data set and to increase the relevance of the selected sources, the proceedings papers were excluded (directly at WoS, resulting in 221 papers). Next, all abstracts were carefully read in order to perform a more “high level” filtering, excluding papers that were related to surge arresters and CBM but could be considered (somehow) out of the scope of this review (for example, papers related to CBM of high-voltage equipment in general or papers more related to insulation coordination, etc.). Following the “high level” filtering, the dataset has been reduced to 111 papers. Figure 4 presents the evolution per year in the number of publications and citations related with to the CBM of surge arresters (generated directly at WoS). As the search was performed in July of 2023, the data from the year 2023 are still incomplete. It is possible to notice a steady growth in interest in this subject.

**Figure 5:**
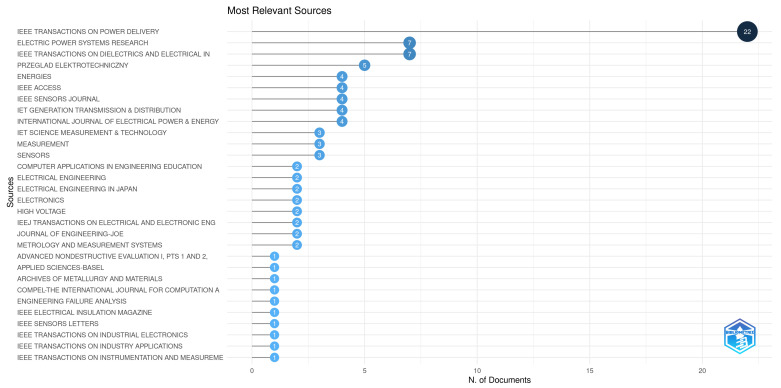
Most relevant journals for publishing in the field of CBM of surge arresters—Figure generated in the Bibliometrix package for R.

The metadata (with information such as keywords, citations, authors, affiliations, publishing journals, etc.) from the 111 selected papers have been exported to a text file in order to perform the bibliometric analysis (using third party softwares such as the Bibliometrix package for R [39] and the VOSviewer [40]—both free software and open source). Using these software, interesting quantitative information can be extracted from this group of papers. Figure 5 presents the most relevant sources (or the journals with the most appearances within this group). It is very interesting to notice that the journals on top of this list are more related to power systems in general (IEEE’s *Trans. on Power Delivery* and *Trans. on Dielectrics and Electrical Insulation*, Elsevier’s *Electric Power Systems Research* and MDPI’s *Energies*) rather than those more related to sensors and instrumentation (IEEE’s *Sensors Journal* and *Trans. on Instrumentation and Measurement*, IET’s *Science and Measurement & Technology*, *Elsevier*’s *Measurement* and MDPI’s *Sensors*)—which, in fact, represents opportunities to be explored in this latter segment.

**Figure 6:**
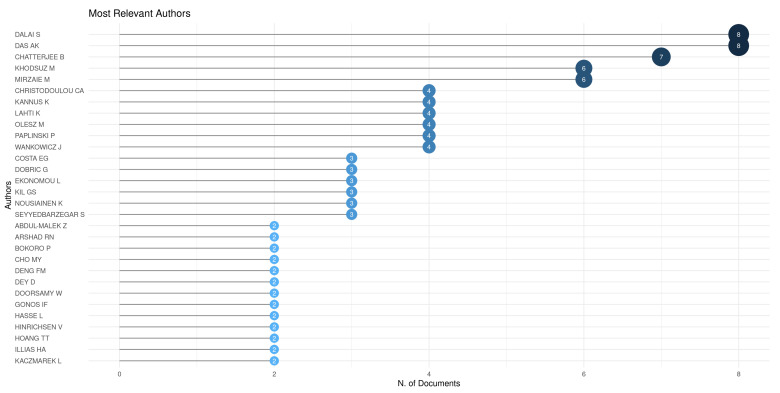
Most productive authors in the field of CBM of surge arresters—Figure generated in the Bibliometrix package for R.

Figure 6 presents the most relevant authors (or the authors with higher productivity within the analyzed group of papers). It is worth mentioning the high productivity (in this field) of *Arup Kumar Das* and *Sovan Dalai* (both from *Jadvpur University*, India), *Soumya Chatterjee* (from the *National Institute of Technology Durgapur*, India—which also has collaborations with *A.K. Das* and *S. Dalai*), *Masume Khodsuz* (from *University of Science and Technology of Mazandaran*, Iran) and *Mohammad Mirzaie* (from *Babol Noshirvani University of Technology*, Iran—which also has collaborations with *M. Khodsuz*). In fact, all authors appearing in this list present potential for collaborations (projects, graduate studies, etc.) in this field of research.

**Figure 7:**
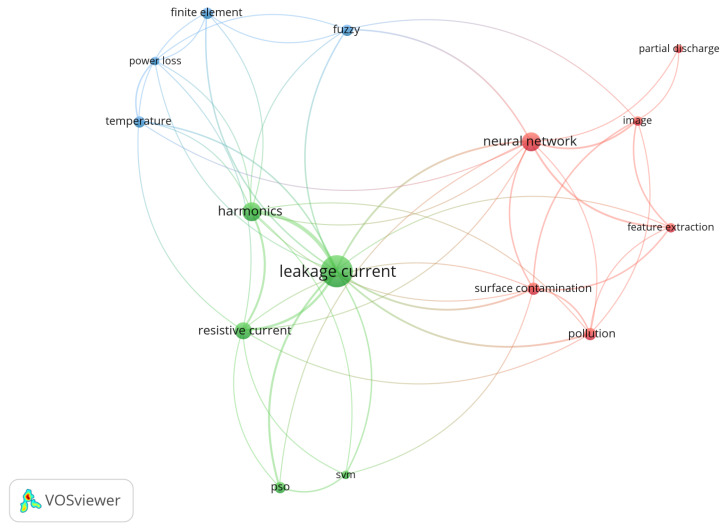
Co-occurrence of keywords (minimum of 3 occurrences)—Figure generated in VOSviewer.

A co-occurrence chart presents relationships between the keywords. It identifies keywords that appear together in different papers. Figure 7 presents a co-occurrence chart for the keywords that appear together (with a minimum of three occurrences in different papers). It can be noticed that the papers that discuss “leakage current” also discuss “resistive current” and “harmonics” (mainly because the software found a strong co-occurrence between these keywords and placed them close together). Other keywords, such as “neural network” are also related to “leakage current” (as there is a connection line between them), although in this analysis “neural network” is more closely related to “feature extraction” and “image”, for example. It is important to note that this type of analysis is the result of a computer software. It serves to give an overview of a study field, although a more in-depth literature review (such as the one presented in Section 3.2) is still required in order to better understand the subject.

**Figure 8:**
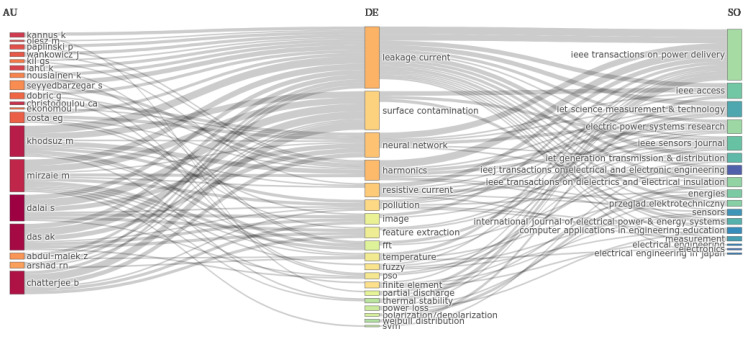
Relations between keywords (center) with most productive authors (left) and most relevant journals (right)—Figure generated in the Bibliometrix package for R.

Figure 8 presents a three-field plot, showing the relations between the keywords (center) with the most productive authors (left) and the most relevant journals (right). This type of visualization allows us to easily identify what authors have contributed to a given subject (keyword) and what journals are more active in that subject.

**Figure 9:**
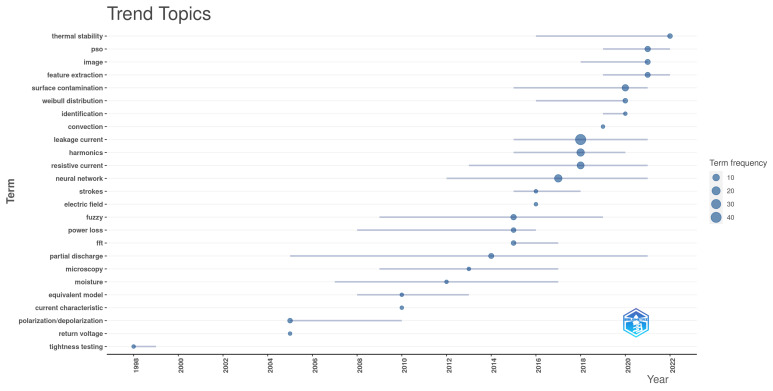
Trend topics—Figure generated in the Bibliometrix package for R.

Finally, the bibliometric analysis can be useful for identifying trend topics. Figure 9 presents the trend topics plot, where the keywords are distributed over time according to their popularity in a given year. It can be noticed that methods such as “polarization/depolarization” and “return voltage” were more frequent in the past, while more recent studies are more focused on “feature extraction” using machine learning/artificial intelligence techniques such as “PSO” or “neural networks”, for example.

### 3.2. Comprehensive Literature Review

The most popular techniques for monitoring the condition of MOSAs are based on leakage current and temperature. Leakage current and analysis of its harmonic content is based on the IEC 60099-5 standard [41]. Temperature monitoring has become popular due to its practicality of execution with the equipment in operation [42]. Other methodologies presented in the literature monitor the other parameters discussed previously in Section 2.2, Section 2.3, Section 2.4, Section 2.5, Section 2.6, Section 2.7, Section 2.8, Section 2.9, Section 2.10 and Section 2.11.

#### 3.2.1. Methods for Monitoring the Leakage Current

**Figure 10:**
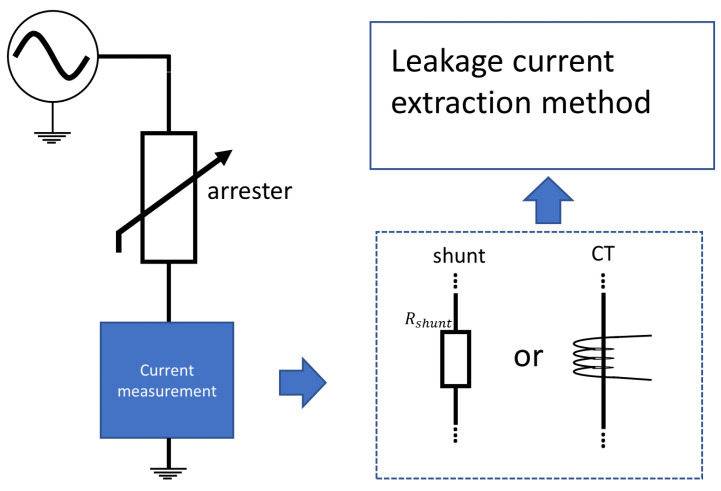
Measurement diagram for monitoring the leakage current.

Figure 10 presents the measurement diagram for monitoring the total leakage current. This measurement can be performed through a shunt resistor (which can be considered invasive) or using current transformers (which can be considered non-invasive). After obtaining the signals, it is possible (through techniques described below) to separate the capacitive component from the resistive component of the total leakage current.

The modification in the waveform of the leakage current, as well as its magnitude, are indicative of degradation in arresters. For greater effectiveness, it is necessary, according to IEC 60099-5 [34], to analyze the resistive component and use the applied voltage as a reference for extracting the component. The use of the total leakage current becomes cheaper, but it may mean nothing or very little. Abdullah et al., in [43], developed a guideline for a 33 kV network, where a total leakage current below 500 μA has been considered normal; between 500 μA and 1.5 mA has been considered cautious; and above 1.5 mA has been considered critical. Also, an interesting application of the total current is presented by Voncina et al. [44], as they describe the hardware details of an electronic device to detect the resistive component (from the total current) without measuring the voltage.

The literature presents techniques for extracting the resistive component of the leakage current [13,14,15,44,45]. The method presented by Abdul-Malek and Aulia [13] introduces the Modified Shifted Current Method (MSCM) technique, which eliminates the use of a reference voltage to extract the resistive component. The method presents high accuracy compared to the Capacitive Current Compensation Method (CCCM) [14,28], but does not consider the harmonics introduced by the voltage (which end up being attributed to the behavior of the leakage current). Khodsuz and Mirzaie [45] introduce an improvement in the time-delay addition method (TDAM), where the effects of harmonic components from the voltage waveform are removed (giving it the name ITDAM—Improved Time-Delay Addition Method). In the work of Xu et al. [15], the Current Orthogonality Method (COM) is used. It has good noise immunity and accuracy, but its performance is affected by the presence of harmonic content in the supply voltage.

The analysis of the frequency components of the current waveform is an indicator of the condition of the arrester. The current analysis present in the literature is based on the use of the Fast Fourier Transform (FFT) algorithm to obtain the harmonics of the leakage current. Stojanovic and Stojkovic [46] presents a comparison of the effectiveness of monitoring of total leakage current, the resistive component and the power loss when the terminal voltage is distorted. The fifth-, seventh-, and ninth-order voltage harmonics affect the reliability of the analysis of isolated third-order harmonics. Only the measurement of leakage current appears to be ineffective, given the strong influence of harmonics in the voltage. Munir et al. [47] proposes the use of the fifth-order harmonic component of the resistive leakage current as an indicator of ageing. It is presented that the variation of the fifth-order resistive leakage current with the fifth-order voltage is smaller than the variation of the third-order resistive leakage current with the third-order voltage. Khodsuz et al. [48] addresses the evaluation of the influence of various factors, including overvoltage and voltage harmonics. It is concluded that the first-order capacitive component allows differentiating changes in leakage current resistance caused by voltage fluctuations or aging of the arrester.

The references presented above show the dependence on the knowledge of the voltage waveform. According to [49], this is an important limitation, since there are practical and safety restrictions on the measurement of high voltages in the field. Lee and Kang in [23] propose a method based on time delay that allows the extraction of the resistive component of the leakage current without measuring the voltage across the arrester. Some techniques are based on the use of the total leakage current (although the IEC 60099-5 standard [41] states that the measurement of the total current is “less sensitive and often not meaningful”)—hence, there is no need to measure the terminal voltage in order to extract the resistive component. Metwally et al. [22] propose using Prony analysis on the total current in order to overcome the issues related to FFT (such as spectral leakage, aliasing and the ability to handle nonstationary signals). Lira and Costa in [49] proposes the processing of the total leakage current with an artificial Multi Layer Perceptron (MLP). Other similar approaches based on Machine Learning (ML)/Artificial Intelligence (AI) have been presented by Hoang et al. [50] and Chen et al. [51] (where Particle Swarm Optimization (PSO) is used in a Support Vector Machine (SVM) classifier), by Gil et al. [52] (where a k-nearest neighbors (KNN) algorithm is used) and by Dobrić et al. [53] (where a Genetic Algorithm (GA) is used).

Recently, Haider et al., in [54], presented a new method based on a correlation between the power factor and the total current. The power factor is obtained based on the MSCM (which does not require the acquisition of terminal voltages in order to extract the capacitive current of the total current). The authors claimed improved computational efficiency, compared to previous methods. The authors, however, have not analyzed the performance of their method in case of harmonics in the voltage (which was one of the issues with the MSCM).

#### 3.2.2. Methods for Monitoring the Temperature

The thermal behavior of the arrester reveals its condition. Thermography gained prominence in the area of predictive and preventive maintenance of electrical equipment in general [55]. In the area of surge arrester condition monitoring, an increase in operating temperature indicates damage or degradation [24]. The literature presents several works applying the thermography maintenance technique, either applied alone or in conjunction with leakage current monitoring and the use of artificial intelligence (AI) techniques to provide diagnosis.

**Figure 11:**
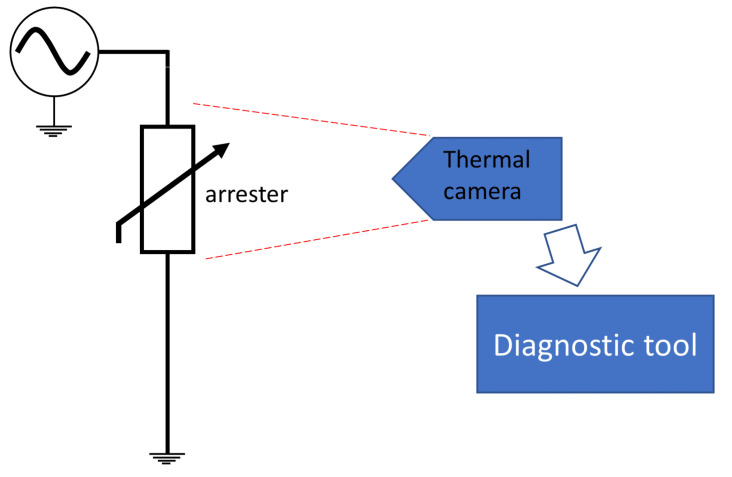
Measurement diagram for monitoring the temperature.

Figure 11 presents the measurement diagram for monitoring the temperature. It is based on recording thermal images of the arresters. The obtained data undergo visual analysis (by a human, carried out on site) or are sent to a computer and processed with the help of AI algorithms.

The use of pattern recognition in thermal images is presented in the literature with the application of classifiers. In the work of Wanderley Neto et al. [24], thermal patterns are classified and identified for different types of degradation and failures using an artificial neural network (ANN). In the work of Das et al. [30] a convolutional neural network (CNN) is applied to classify thermal images of arresters, allowing the identification of severe pollution levels. Also, in the work of Laurentys Almeida et al. [42], a neuro-fuzzy classifier is applied in thermovision data.

Applications of machine learning classifiers aim to identify changes and modifications in images that are imperceptible to human visual analysis. These applications have the ability to handle high-dimensional and multivariate data and to extract implicit relationships within large data sets [56]. Machine learning provides powerful approaches for predictive maintenance, although the performances are dependent on choosing an ML technique [57]. In Novizon and Abdul-Malek [58], the use of an MLP neural network is presented, which, through correlation of features extracted from the leakage current and thermal image, allows the classification of the operational state of the arrester. The combined use of indicators presented in the literature is highly effective. Also, in the work of Huang and Hsieh [59], a regression model combining temperature monitoring and extraction of resistive leakage current characteristics is presented.

#### 3.2.3. Methods for Monitoring Partial Discharge

Partial discharges can be caused by high-voltage stresses and may result in increased maintenance costs [60]. The monitoring and diagnosis of partial discharges is being widely adopted in electrical plants [61]. Initially, there were several obstacles that impacted its reliability as an online monitoring tool [62]. Advances in the area allowed an increase in the immunity to electrical noise of monitoring systems for online use. Partial discharge (PD) monitoring is used in high-voltage equipment; for example, high-voltage rotating machines [62], transformers [63] and power cables [64]. The methodologies for detecting partial discharges can be carried out through acoustic emissions [65] or UHF sensors [63].

**Figure 12:**
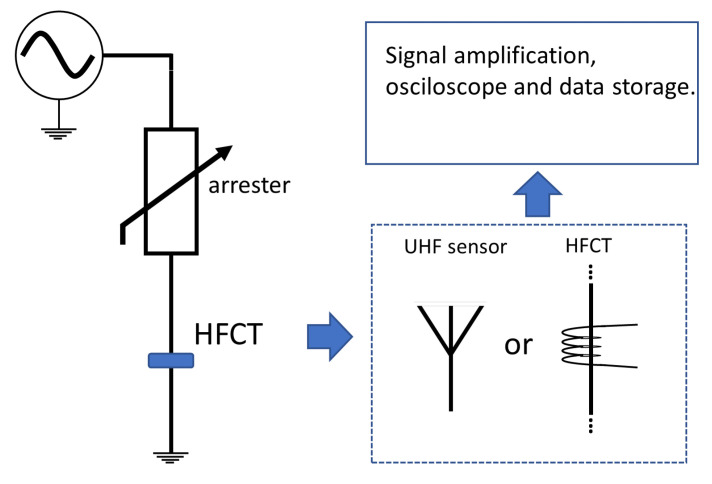
Measurement diagram for monitoring partial discharges.

Although partial discharges are generally measured in insulators and high-voltage equipment, the case of arresters is no different. In this case, its occurrence can compromise the impermeability of the casing, an essential characteristic that guarantees the longevity of the arrester in field operations [27]. Figure 12 illustrates the general concept through measurement of partial discharges. Wong, in [66], proposed a system for capturing electromagnetic emissions composed of a biconical antenna and using Wigner–Ville Distribution (WVD), generating a two-dimensional time/frequency image as a result. In their work, Amorim Jr. et al. [67] used a high-frequency current transformer (HFCT) sensor for online monitoring of partial discharges. Another work addressing the use of HFCT is presented by Tatizawa et al. in [68].

As observed for temperature and current monitoring techniques, methodologies combining PD and leakage current monitoring appear in the literature. In [69], Shihab et al. proposed a system combining partial discharge monitoring with leakage current monitoring in order to detect the effects of pollution on an outdoor plant.

Studies relating PD activity of arresters in the presence of aggressors in the field are also presented in the literature. However, as reported by da Silva et al. in [70], the ability to detect moisture penetration inside the arrester via partial discharges seemed not to be as sensitive as the power loss methodology.

#### 3.2.4. Methods for Monitoring Power Loss

**Figure 13:**
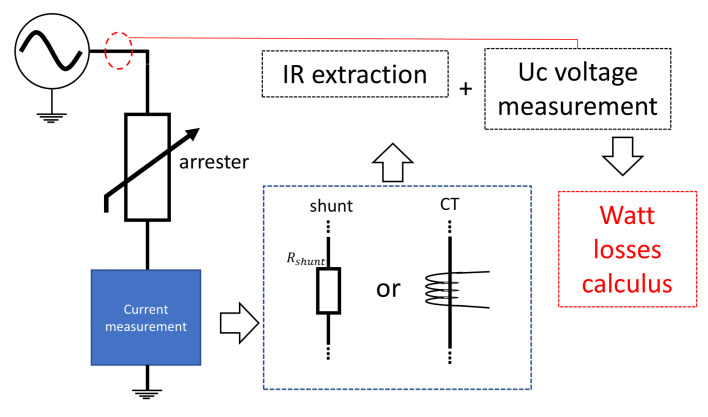
Measurement diagram for the power loss method.

The power loss in the arrester has a direct correlation with the reduction of their useful life. According to a comparative table presented by Abdul-Malek et al. [9], the power loss method has high sensitivity to the arrester health and is very informative and very versatile, although it has a high initial cost and is not easy to use. Figure 13 presents a general diagram for the measurement of power loss. The method requires measurement and extraction of the resistive leakage current and also requires measurement of the terminal voltage of the arrester. The calculation to determine the active power dissipation is performed using Equation (4).

(4) $$P=\frac{1}{T}\int_{0}^{T}u_{c}\left(t\right)\cdot i_{R}\left(t\right)\cdot dt\;,$$

where *T* is the integration period (referring to one fundamental cycle), $u_{c}\left(t\right)$ is the terminal voltage and $i_{R}\left(t\right)$ is the resistive leakage current.

In Miller et al. [29] , the power loss method is employed in order to compare different types of polymer housing of arresters (under climate conditions of coastal Florida). Their study concludes that the power loss is dependent not only on the varistor blocks (internal loss) but also on the housing (external loss) and that non-silicone housing presented external losses 10 times higher than their internal losses (considering surface contamination).

In field situations, the problems encountered in relation to obtaining power loss include measuring the terminal voltage of the arrester. Klein et al., in [71], implemented a monitoring system based on measuring current and power loss. The difficulties highlighted refer to high-voltage measurement methods (described as expensive and in some cases difficult to access).

#### 3.2.5. Methods for Monitoring Insulation Resistance

**Figure 14:**
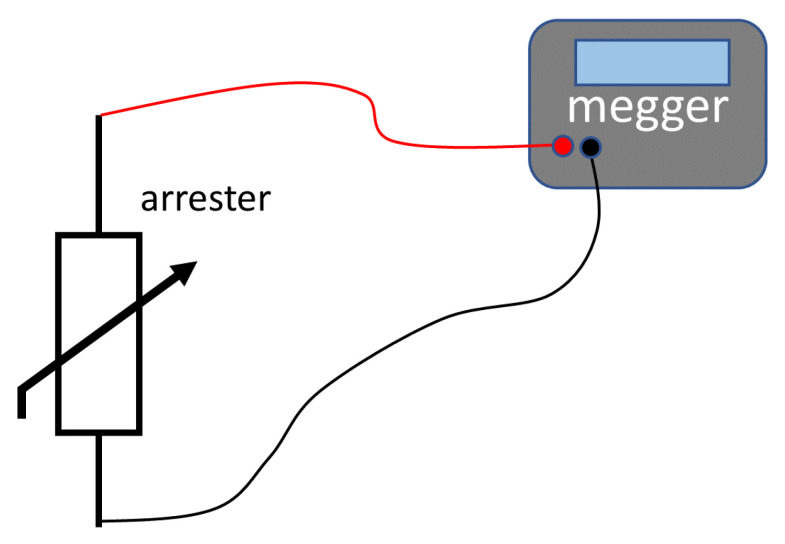
Measurement diagram for the insulation resistance method (megger).

The monitoring of the insulation resistance (with a megger equipment) is popular in the industrial environment, with applications for insulators [72], insulation of rotating machines [73] and transformers [74]. The behavior of the arrester under normal conditions is equivalent to that of an insulator. Figure 14 presents the general measurement diagram for the insulation resistance method using a megger. The arrester has to be removed from operation and a DC voltage is applied to its terminals. After several minutes (in order to remove the capacitive and polarization effect on the measured DC current), the equipment displays the insulation resistance. In [75], Uchida et al. presented the use of a megger for evaluating oil-immersed ZnO surge arresters (in conjunction with measurement of reference voltage and monitoring of leakage current). Their results show a decrease in insulation resistance after forced degradation. However, according to El-Rasheed [76], the megger test is only suitable for low and medium voltage arresters [76].

#### 3.2.6. Methods Based on Reference Voltage

**Figure 15:**
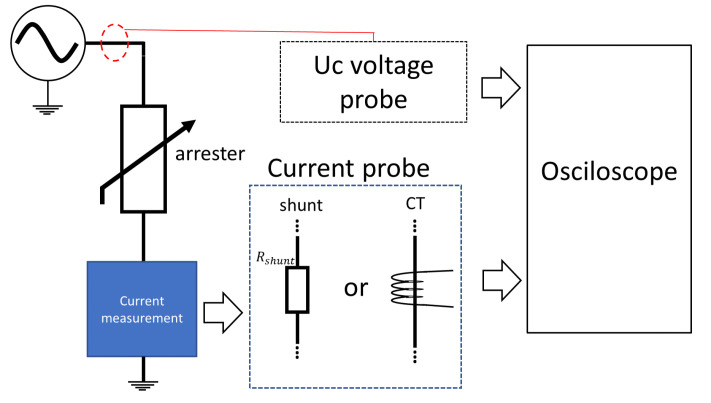
Measurement diagram for the reference voltage method.

Reference voltage monitoring is a technique that can only be performed in laboratory environments. The need to regulate the voltage applied to the arrester terminals makes the methodology inapplicable in the field. Figure 15 presents the general diagram for the reference voltage method. The laboratory voltage is measured with the aid of a high-voltage probe and is adjusted until a resistive leakage current is obtained (measured with shunt or CT, at values between 0.05 mA and 1 mA per square centimeter of the disk area).

Schichimiya et al. [77] presented the development of an advanced arrester for application in gas-insulated substations (GIS) using the reference voltage technique as a way of evaluating the capabilities of the developed equipment. The use of the reference voltage methodology is also applied in the literature as a way of analyzing and validating other methodologies. Uchida et al. [75] applied the technique to validate the monitoring methodology using meggers and leakage current. The reference voltage measurement is also commonly applied to verify the degradation after an impulse test. Koga et al. [78] use it to study the degradation caused by the applied impulse currents and the loss of the ability to extinguish the discharge current in arresters with a gap.

#### 3.2.7. Methods Based on Surge Counting

Monitoring the number of discharges is not an efficient indicator of degradation. Quantifying the number of events without considering the amount of energy dissipated by the arrester does not provide a reliable diagnosis. The diversity between atmospheric discharges when considering the peak value, front time, tail time, and amount of energy makes the method unreliable. Counters are inefficient at capturing multiple discharges, where the interval between discharges is less than 50 ms [8]. Also, Tiechen and Bo in [79] reported an excessive counting number in a gas-insulated substation (GIS), in which the susceptibility of the counters to pulses due to stray capacitances was identified. There are, however, studies in the literature that use the number of discharge incidents as part of diagnostic tools in order to predict the need to replace the arrester. In their work, Araujo et al. [80] presented a methodology for predicting the need to replace distribution arresters based on the correlation between faults in the system and the incidence of atmospheric discharges measured by discharge counters (as well as other measurements). Also, Wen et al. [81] proposed an image processing system for extracting parameters from leakage current meters in conjunction with surge counters installed in the field.

#### 3.2.8. Methods Based on Residual Voltage

**Figure 16:**
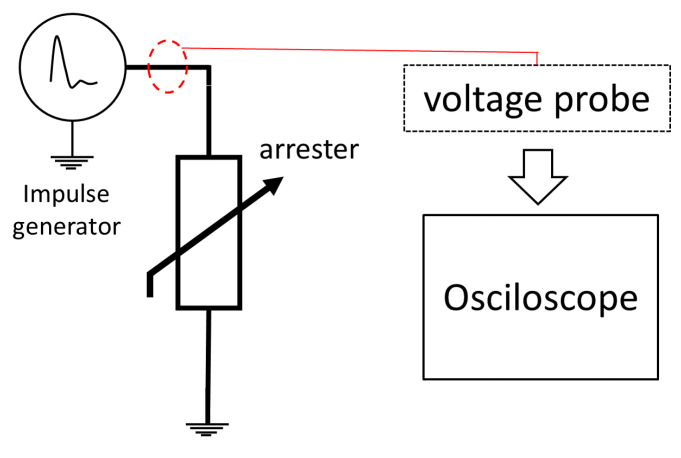
Measurement diagram for the residual voltage method.

The use of the residual voltage measurement is applied in the development of models to simulate the transient response of arresters and also as a way of analyzing them (offline, in laboratories). Figure 16 presents the general measurement diagram for the residual voltage method. Current impulses are applied to the arrester and the voltage drop across its terminals are measured. The observed signal would represent the behavior of the tested arrester when a discharge occurs. Kannus and Lahti, in [82] have analyzed a significant number of arresters (including MOSA and SiC), between operating ages of 4 to 38 years. As one of their tests, impulses have been applied and the residual voltages have been measured. Due to its transient nature, residual voltage has a short duration. This implies that there is a need to measure high-frequency signals and requires certain care (appropriate instruments and probes) in order to guarantee the integrity of the signals. The occurrence of reflections and other electrical effects modifies and inserts information into the signals that may not be real or may remove real information. In their work, Metwally [83,84] demonstrated changes caused by commercial voltage dividers in the residual voltage waveform and presented the development of a measuring probe which removes these changes.

The application of AI is also present in the residual voltage methodology. In their work, Yutthagowith et al. [85], presented a model based on a genetic algorithm in order to simulate the residual voltage of the arrester. This model assists the engineering area in selecting and configuring load voltages to generate the desired current waveform.

#### 3.2.9. Methods Based on Dielectric Polarization

**Figure 17:**
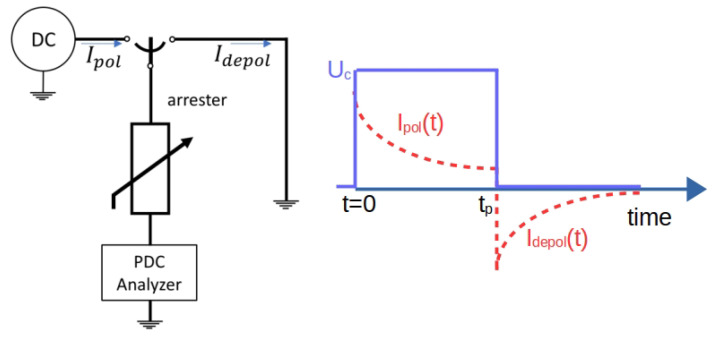
Measurement diagram for the polarization/depolarization current method—based on [17].

The arrester behaves like a dielectric under normal operating conditions, which makes measurement methodologies for insulating materials applicable in the area of arrester maintenance. In the literature, two techniques are presented that derive from the dielectric polarization effect, namely Return Voltage and Polarization/Depolarization Current PDC. Figure 17 presents the general measurement diagram for the PDC method. A voltage step is applied to the sample and its polarization current is measured. After a time interval $t_{p}$, the sample is discharged (represented in the figure as the switching position of the connections) and the depolarization current is measured. Based on the difference between these currents, the conductivity of the sample can be determined, as presented by Equation (5) [17]).

(5) $$\sigma \approx \frac{\epsilon_{0}}{C_{0}\cdot U_{c}}\cdot \left[i_{pol}\left(t\right)-i_{depol}\left(t\right)\right]\;,$$

where $\epsilon_{0}$ is the vacuum dielectric permittivity, $U_{c}$ is the value of the voltage step and $C_{0}$ is the measured value of capacitance of the sample.

Mardira et al., in their comparative study [7] (which also included the the reference voltage method and the residual voltage meyhod), demonstrated that both the return voltage measurement and the polarization/depolarization current methods are good indicators of MOSAs degradation. However, Saha and Mardira stated in [86] that “results from these diagnostics cannot be interpreted confidently unless the exact relationship between the measured dielectric parameters and the fundamental dielectric processes of insulations is clearly understood”. Hence, an insulation model for the arrester (consisting of RC branches) is proposed in [86], based on the polarization/depolarization current. In their study, Bassi and Tatizawa [17] have extended this model to more branches, achieving a better fit. Also, ref. [86] detected early degradation of the arrester (for few applied impulses) while the reference voltage and residual voltage methods did not. Complementing [86], ref. [17] has applied more impulses (until the catastrophic failure of the samples) and detected that both the reference voltage and the residual voltage methods do not present expressive variations until the actual failure.

#### 3.2.10. Destructive Methods

Destructive methods are applied to observe the microstructure of ZnO varistors. Knowledge of the chemical and microstructural behavior of ZnO blocks allows a better understanding in the area of developing new varistor elements and the influences of different factors on the microstructure. Changes in the microstructure of aged varistor blocks demonstrate the effects caused by the degradation process [87]. The chemical differences in microstructures result in different degradation patterns [88]. The techniques that are applied to analyze the microstructure are divided between Optical microscopy (OM), Scanning electron microscopy (SEM), X-ray diffraction (XRD) and Energy-dispersive spectrometry (EDS) [7].

In their study, Bokoro and Doorsamy [88] presented an experimental statistical design methodology, based on the use of SEM, in order to evaluate the influence of microstructural composition and a distorted voltage supply on the degradation of the arresters. In their work, de Salles et al. [87] also investigated the effect of operating temperature on degradation caused by current impulses. The authors pointed out the appearance of voids in the microstructure of the varistors (observed by the SEM technique), as well as the formation of pyrochlore in the ZnO block identified using EDS and XRD techniques. Another approach, using only the OM method, is presented by Paplinski et al. [36] , who evaluated the microstructure and susceptibility to thermal and electrical stresses in field conditions.

Besides aging and degradation of the microstructure, physicochemical tests can also provide other important information. The control of formation of the microstructure can also guarantee improved electrical properties of varistors. Izoulet et al. [89] and Simo et al. [90] both made improvements to the manufacturing process of the varistor blocks that allowed increases in the nonlinearity coefficient of the ZnO blocks and a reduction in the leakage current. Also, in their work, Litzbarski et al. [37], combined measurements of electrical parameters and physicochemical tests in order to measure the repeatability in the fabrication process of ZnO varistors, for production quality purposes.

#### 3.2.11. Resonant Ultrasound Spectroscopy—RUS

**Figure 18:**
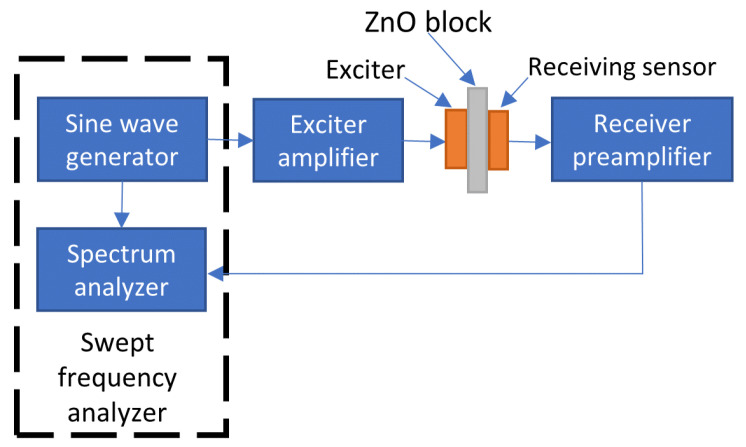
Measurement diagram of RUS method—based on [91].

Resonant Ultrasound Spectroscopy (RUS) is a technique that consists of subjecting the material under test to sound signals at different frequencies (performing a frequency sweep). RUS makes it possible to study the elastic properties of various materials and measure the natural frequencies of vibrations [91]. Figure 18 presents a measurement diagram of the RUS method, in which the output of a sine signal generator is amplified and applied to a piezoelectric exciter. The exciter is in direct contact with the arrester (ZnO block). At the opposite side of the arrester, there is a receiver sensor. The received signal is then amplified and fed back to the equipment for the spectral analysis. Hasse et al., in [91,92] performed the RUS in order to verify the quality of ZnO blocks during the manufacturing process (before the metallization).

#### 3.2.12. Frequency Response Analysis—FRA

Frequency Response Analysis (FRA) is an offline technique widely used for offline diagnostics of power transformers. It consists of the comparison of two spectra (of an electrical parameter of the device under test) measured at different instants of the lifetime of the device and differences between the spectra may indicate the onset of a failure [93]. Apart from its use in transformers (in which case its use is guided by the standard IEEE-Std-C57.149 [94]; several types of commercial equipment are available in the market for this purpose [95]) some other innovative applications for FRA have been proposed as well. Among them, recently, Zacarias et al. [96] proposed using FRA to detect failures in arresters. The presented results, although preliminary, have shown that FRA was capable of detecting early damage in arresters.

**Figure 19:**
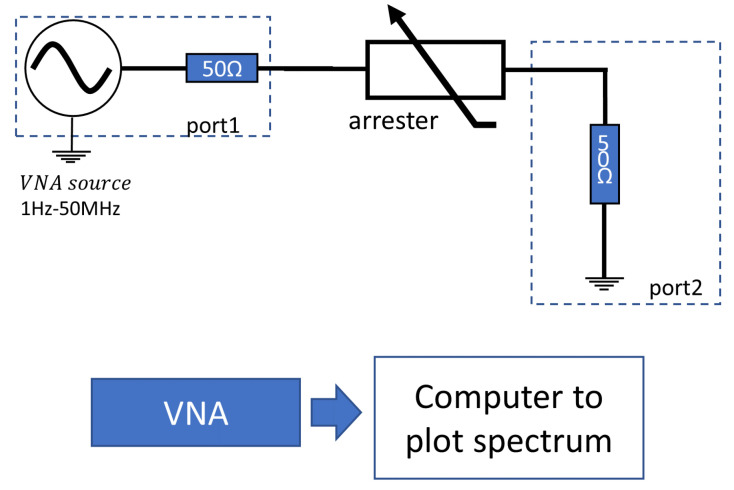
Measurement diagram of FRA method considering the Bode-100 equipment in the Series-Thru connection.

The commercial FRA equipment are based on a Vector Network Analyzer (VNA) and, usually, have several possibilities for circuit connection. Figure 19 presents a type of connection called “Series-Thru” in the Bode-100 equipment (of manufacturer OMICRON Lab) [97]. For each frequency injected by the VNA, an impedance value is calculated using Equation (6), valid for the Series-Thru connection. An impedance spectrum is then generated (considering a wide range of frequencies) and the comparisons between spectra may indicate a degradation condition.

(6) $$Z=100\times \frac{1-S_{21}}{S_{21}}\;,$$

where $S_{21}$ is the voltage gain between ports 2 and 1 indicated in Figure 19.

#### 3.2.13. Known Measurement Issues of the Methods

The literature also discusses measurement issues and inaccuracies of some of the presented methods. The measurement of leakage current (and the extraction of its components) is susceptible to errors due to various factors. In their study, Olesz et al. [98] presented an experimental analysis of the errors, depending on the type of measurement used to obtain the leakage current and terminal voltage of the arresters. For the leakage current, it was found that the use of current transformers (CT) causes phase and amplitude errors and a calibration procedure (using a shunt resistor) has been presented. In case of terminal voltage measurement, it was observed that potential transformers (PT) may present distortions and phase delays in voltage signals only at high frequencies (higher than 8 kHz) and no significant errors were found in the frequency range until the 25th harmonic. Recently, Teymourian et al. [99], have analyzed the effect of non-uniform superficial pollution on the harmonic components of the resistive leakage current and concluded that the diagnosis can be impacted.

In case of thermography, the thermal images obtained in-field are sensitive to internal and external factors that can influence the measured temperatures [100]. Ursine et al. [100] presented experiments to evaluate the influence of these factors divided between solar radiation, wind speed, temperature and parameters of the thermal camera. Solar radiation directly impacted the measurements and in some cases may provide a false diagnosis. The same is observed for variations in wind speed. Concerning the parameters of the thermal camera, the emissivity presented a considerable influence on the displayed temperature—an error in the choice of this parameter can compromise the entire diagnosis. Also, it was reported by Gasiyarov et al. [101] that superficial pollution may cause increased heating, not in the internal varistors, but on the ribs of the arrester.

In-field measurement of partial discharges is susceptible to electromagnetic interference. The PD signal can be superimposed by noise originating from the environment surrounding the PD source and measurement equipment [102]. The use of HFCT and UHF requires the application of noise-removal techniques (Discrete Wavelet transform, FFT and ANN). In contrast, there are acoustic methods that have high immunity to electromagnetic interference, but need to be directed to the PD source, as stated by Refaat and Shams in [102].

Power loss measurement is widely used in laboratories but rarely on-site [20] due to the difficulty of measuring high voltages. The same issues with CTs and PTs, previously discussed for leakage current, are also expected.

Concerning the Residual Voltage method, due to the high-frequency nature of the applied impulses, the measured signals may be affected by the measurement circuit. Metwally [83] presented an analysis of residual voltage signals measured by commercial probes and detected inaccuracies, such as a longer rise time, initial overshoot superimposed and a decay in the voltage before the peak in the impulse current.

#### 3.2.14. Comparison of Methods

**Figure 20:**
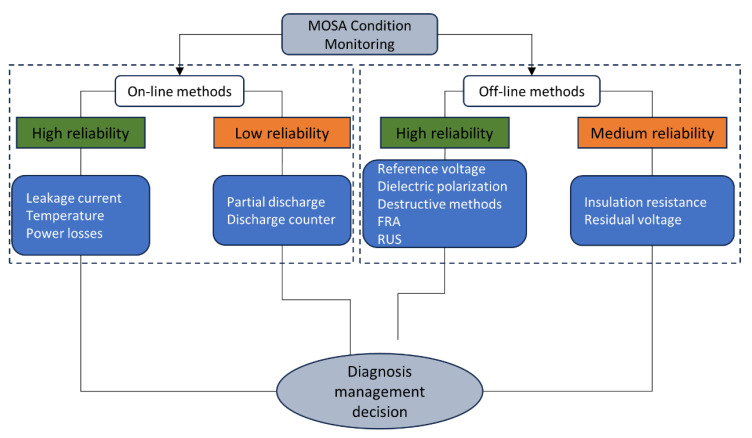
Classification of the previous methods according to their applicability and reliability.

Based on the information provided in the literature (mainly in the comparative tables provided in [9]), this subsection provides a comparative summary of the methods, as Table 1 and visually as Figure 20.

## 4. Conclusions

This article presented a bibliometric and comprehensive review of the literature on monitoring methodologies for metal oxide arresters.

Using the open-source softwares Bibliometrix (used within R) and VOSviewer, the bibliometric analysis has been performed on exported data from the database Web of Science (which includes papers from IEEE, MDPI, Elsevier, etc.). The goal of this analysis was to gain insight into the literature prior to the more in-depth comprehensive research. The analysis revealed the most relevant journals to publish in this field, the most productive authors (which is interesting for the prospect of future collaborations), the most important keywords (related to the monitoring methods) and how these keywords relate to each other and relate to the most productive authors and the most relevant journals. The analysis also revealed how these keywords appeared in the literature over the years, which is useful in order to make predictions about future trends for research.

It is important to highlight that the bibliometric analysis is based on quantitative results produced by computer software. It serves to provide an overview of a study field, although more in-depth, comprehensive research is still required.

This paper has also presented a traditional more in-depth comprehensive study. Both online and offline methods have been qualitatively analyzed. Whenever available in the literature, the drawbacks of some variations of a given method has been reported as well as measures that have been implemented in the literature in order to mitigate that drawback. The presented literature review is also up to date and includes the recent development of the application of the FRA technique on arresters. In order to present a quick comparison between methods, a table presenting the advantages and disadvantages of each method has been presented. The known measurement issues and inaccuracies of the methods have also been presented.

It has been detected that the two most widely used methods (variations that analyze the leakage current and variations that use thermography) have issues concerning superficial pollution. This finding can also be extended to other online methods, as the superficial pollution has an impact on the electrical properties. On the other hand, offline methods can be immune to pollution, as the samples are usually cleaned before the measurements. Also, some offline methods analyze the microstructure of the varistors directly; these methods are the most accurate. It is important to note that the choice between methods relies on several particularities of the system in which the arresters are in use. Also, as indicated by the bibliometric analysis, there is a recent trend in methods that employ some form of Artificial Intelligence. A very welcome feature of these methods could be the detection of superficial pollution and some form of compensation for the diagnosis.

## Figures and Tables

**Table 1:** Comparison of methods.

Method	Advantages	Disadvantages	Application Area	Reliability
Leakage Current	Directly related to the degradation condition of the arrester. It can be carried out with the arrester in service.	Susceptible to the presence of voltage harmonics and voltage fluctuations.	Laboratory and In-Field.	High.
Temperature	Directly related with the degradation condition of the arrester. It can be carried out with the arrester in service. Non-invasive.	Susceptible to climatic factors (wind speed, solar radiation, etc.)	Laboratory and In-Field.	High.
Partial Discharge	Non-invasive.	Susceptible to electromagnetic interference.	Laboratory and In-Field.	Low.
Power Loss	Directly related with the degradation condition of the arrester. Not affected by harmonics.	Difficulty for In-Field application (requires measurement of terminal voltage).	Laboratory and In-Field.	High.
Insulation Resistance	Safe for the maintenance operator.	Cannot be performed In-Service. Only suitable for low- and medium-voltage arresters.	Laboratory.	Medium.
Reference Voltage	Directly related to the degradation condition of the arrester.	Requires test voltages higher than the nominal. Cannot be performed In-Service.	Laboratory.	High.
Surge Counting	Displays measurement of leakage current.	Does not measure the energy of the discharges nor provides any information about the discharges.	In-Field.	Low.
Residual Voltage	Displays the arrester’s response to a discharge.	Cannot be performed In-Service.	Laboratory.	Medium.
Dielectric Polarization	Directly related with the degradation condition of the arrester.	Cannot be performed In-Service.	Laboratory.	High.
Destructive Methods	Provide details on the physicochemical characteristics of the varistors.	Are destructive to the samples. Cannot be performed In-Service.	Laboratory.	High.
RUS	Provide some properties of the microstructure in a non-destructive way.	Cannot be performed In-Service.	Laboratory.	High.
FRA	Electrical parameters can be obtained from the spectra.	Cannot be performed In-Service.	Laboratory.	High.

## Data Availability

Data sharing is not applicable to this article.

## Abbreviations

The following abbreviations are used in this manuscript:

AI	Artificial Intelligence.
ANN	Artificial Neural Network.
CBM	Condition-Based Maintenance.
CCCM	Capacitive Current Compensation Method.
CNN	Convolutional Neural Network.
COM	Current Orthogonality Method.
CT	Current Transformer.
EDS	Energy Dispersive Spectrometry.
FFT	Fast Fourier Transform.
FRA	Frequency Response Analysis.
GA	Genetic Algorithm.
GIS	Gas-Insulated Substations.
HFCT	High-Frequency Current Transformer.
ITDAM	Improved Time-Delay Addition Method.
KNN	K-Nearest Neighbors.
ML	Machine Learning.
MLP	MultiLayer Perceptron.
MOSA	Metal Oxide Surge Arrester.
MSCM	Modified Shifted Current Method.
OM	Optical Microscopy.
PD	Partial Discharge.
PDC	Polarization Depolarization Current.
PSO	Particle Swarm Optimization.
PT	Potential Transformer.
RUS	Resonant Ultrasound Spectroscopy.
SEM	Scanning electron microscopy.
SiC	Silicon Carbide.
SVM	Support Vector Machine.
TDAM	Time-Delay Addition Method.
UHF	Ultra High Frequency.
VNA	Vector Network Analyzer.
WoS	Web of Science.
WVD	Wigner–Ville Distribution.
XRD	X-Ray Diffraction.
ZnO	Zinc Oxide.
